# Immediate hypersensitivity to common food allergens: an investigation on food sensitization in respiratory allergic patients of Calcutta, India

**DOI:** 10.1097/WOX.0b013e318194c0de

**Published:** 2009-01-15

**Authors:** Jyotshna Mandal, Mahasweta Das, Indrani Roy, Soma Chatterjee, Nimai Chandra Barui, Swati Gupta-Bhattacharya

**Affiliations:** 1 Department of Botany, Division of Palynology and Environmental Biology, Bose Institute, 93/1 Acharya Prafulla Chandra Rd, Calcutta 700 009, India; 2 Surendra Nath College, Calcutta, India; 3 Allergy Department, Institute of Child Health, Calcutta, India

**Keywords:** food allergens, Calcutta, India, skin prick test

## Abstract

**Background:**

Food allergy may be defined as an immunoglobulin E-mediated immune response to food proteins. Such studies have previously not been done in Calcutta, India. The present study was therefore undertaken to record the sensitivity to commonly consumed foods in patients with allergic rhinitis and asthma.

**Materials and methods:**

A survey of 800 patients (410 males and 390 females) reporting to the Allergy Unit of the Institute of Child Health, Calcutta, were selected for the study conducted from May 2006 to April 2007. Respiratory allergic patients in the age group of 5 to 60 years were evaluated using a standard questionnaire, and skin prick test was performed using common food and aeroallergens.

**Results/Conclusions:**

Out of the 684 patients with a history of food allergy, most of them, that is, 338, are in the age group 16 to 40 years, 192 of them were in the age group 41 to 60 years, and 154 were in the age group 5 to 15 years. Most of the patients with food allergy had asthma (65.05%), rhinitis and asthma (20.03%), and skin allergies (4.97%), such as itching, eczema, and urticaria. The foodstuffs that were found to elicit symptoms of hypersensitivity were egg, milk, wheat, pulses, vegetables, fishes, and fruits.

The patients aged between 16 and 40 years (male-female ratio, 1:1.19) were mostly sensitive to prawn, brinjal, banana, ladyfinger, papaya, wheat, and egg. The age group 41 to 60 years (male-female ratio, 1:1.04) had high skin reactivity to brinjal, egg, banana, fish, and *Phaseolus mungo*. Patients younger than 16 years (male-female ratio, 1:1.33) were sensitized to brinjal, prawn, banana, spinach, and egg. We observed that food hypersensitivity also reflects different genetic factors and variations in cultural and dietary habits of each individual.

## 

Food allergy is a major health problem affecting 6% of young children and 3% to 4% of adults [1]. The incidence of perceived food hypersensitivity varies (1.4%-1.9%) largely across different countries probably because of differences in the diagnostic criteria, study design and population variation, and so on [2]. In young children, the common causal food allergens are cow's milk, egg, peanut, wheat, soy, tree nuts, fish, and shellfish, whereas adults mostly have allergies to shellfish, peanuts, tree nuts, and fish [3]. The order of importance of specific allergens varies in different countries, reflecting a possible interaction of genetic factors, cultural and dietary habits, and exposure to new allergenic products early in life [4]. Several food allergies to fruits and nuts have become increasingly common and represent a growing clinical problem [5]. Studies have shown that food allergy in adolescent and adult individuals develops because of an allergic sensitization or cross reaction to inhalant pollen allergens [6,7].

In India, there has been an increase in various allergic diseases from 10% to 30% in the last 4 decades [8]. Many asthma and rhinitis patients may be having food allergy, but only a few studies on food hypersensitivity have been carried out so far in India [9-11]. These studies indicate that common food allergens such as egg, milk, cereals, and legumes induce immunoglobulin E-mediated reactions in children as well as in the adult population [12].

The present study has been designed to determine the incidence of allergic responses toward common food allergens among the respiratory allergy population of Calcutta metropolis, India.

## Materials and methods

### Preparation of Extracts

Various food allergens were defatted in diethyl ether at 4°C. The extraction was carried out by continuous stirring for 8 hours at 4°C in 1:20 wt/vol phosphate buffered saline ([PBS] 0.1 M sodium phosphate, pH 7.2). After centrifugation at 12,000 *g*, the clear supernatant was dialyzed and passed through a 0.22-μm Millipore filter (Millipore Corp, Bedford, Mass). The filtrate was then lyophilized and stored at -70°C in aliquots of known volume in sterile vials.

### Study Subjects

A total of 800 patients (410 males and 390 females) aged 5 to 60 years reporting to the Allergy Unit of the Institute of Child Health, Calcutta, were screened for food hypersensitivity using a standard questionnaire and skin prick test (SPT). The study was conducted from May 2006 to April 2007, and it is composed of patients with a history of bronchial asthma, allergic rhinitis, and urticaria, either alone or in different combinations. The exclusion criteria were perennial or severe asthma, pregnancy or lactation, malignancy, or other severe systemic diseases during skin testing. To avoid masking of severe symptoms, corticosteroids and antihistamines were prohibited. A detailed case history of the subjects was taken based on a structured questionnaire containing information regarding age, sex, religion, occupation, family history, type of diet (vegetarian or nonvegetarian), food habit, smoking habit, onset, duration, and the present status of the symptoms. Patients referred for allergy testing were asked whether they had hypersensitivity symptoms from any foodstuffs listed. Those who answered yes were asked to fill in the questionnaire in which 24 foods were listed along with the other previously mentioned details. The patients were asked specific questions to which they replied whether they had slight, moderate, or severe symptoms, or reported not having eaten that foodstuff. They were also asked to state the symptoms they got from that food hypersensitivity. Food hypersensitivity was ruled out if the foodstuffs had given rise to symptoms only once and did not give rise to any complication if they were repeatedly ingested afterward. Sixty healthy individuals from the city belonging to the same age group were also selected to act as control subjects (confirmed by negative skin reaction). Both male and female patients were categorized into 3 groups: group A (aged 5-15 years), group B (aged 16-40 years), and group C (aged 41-60 years). The study was approved by the ethics committee of the hospital, and informed consents were obtained from the subjects before their participation.

### Skin Prick Test

Skin prick test was performed with a panel of 24 commonly consumed foods--milk, egg, cereals (wheat), pulses *(Lens culinaris, Phaseolus mungo)*, vegetables (brinjal, cabbage, cauliflower, ladyfinger, spinach, tomato), fruits (apple, banana, cucumber, guava, papaya, pumpkin, grapes), meat (beef, chicken, mutton), fish (*Tenualosa ilisha *[locally known as Hilsa], prawn, *Labeo rohita *[locally known as Rohu]), and 12 other common inhalant allergens (pollen and fungi, data not given)--on patients with symptoms suggestive of food allergy (history). Briefly, a drop (20 μL of each extract, 1:50 wt/vol in PBS [wt/vol in glycerinated PBS]) was placed on the forearm, and the skin was pricked with a 26 G hypodermic needle [13]. Histamine diphosphate (1 mg/mL) and PBS (1 mg/mL) were used as positive and negative controls, respectively. The reaction measurement was performed after 20 minutes. According to international guidelines, positivity was defined as a mean wheal diameter of 3 mm or more compared with the negative control [14]. The reaction was graded from + 1 to + 3 level (+ 1, erythema 20 mm in diameter; + 2, erythema > 20 mm in diameter; + 3, wheal and erythema) according to Stytis et al [15]. The skin tests were graded after 20 minutes in comparison with the wheal diameter of the positive control.

## Results

Out of the total number of patients reporting to the clinic, 684 patients (85.5%) were reported to have a history of food allergy, most of them are in the age group 16 to 40 years (n = 338 [49.41%]), followed by 41 to 60 years (n = 192 [28.07%]). One hundred fifty-four (22.51%) belonged to the age group 5 to 15 years. It was observed that most of the patients with food allergy had asthma (17.98%), rhinitis and asthma (74.85%), rhinitis (4.24%), and a small number of patients had skin allergies (1.32%), such as itching, eczema, and urticaria (Table 1). The foodstuffs that were found to elicit symptoms of hypersensitivity were egg, milk, wheat, pulses, vegetables, fishes, and fruits (Table 2).

**Table 1:** Distribution of Patients as Per Symptom and History of Respiratory Allergy

	Total Patients (n = 800; male-female ratio, 410:390)		Patients With a History of Food Allergy (n = 684; male-female ratio, 370:314)	
		
Disease	n	%	n	%
Bronchial asthma	145	18.12	123	17.98
Allergic rhinitis	36	4.5	29	4.24
Asthma with rhinitis	610	76.25	512	74.85
Asthma with skin allergies	12	1.5	9	1.32

**Table 2:** Sensitivity to Different Food Allergens Among the Respiratory Allergic Patients in Various Age Groups

	Age Group, yrs								
	
	5-15, n = 154 (M, 88; F, 66)			16-40, n = 338 (M, 184; F, 154)			41-60, n = 192 (M, 98; F, 94)		
			
Allergens	Total No. Patients Tested	No. Patients Positive (%)	No. Patients Showing > + 1 Level of Reaction (%)	Total No. Patients Tested	No. Patients Positive (%)	No. Patients Showing > + 1 Level of Reaction (%)	Total No. Patients Tested	No. Patients Positive (%)	No. Patients Showing > + 1 Level of Reaction
Wheat (cereal)	138	30 (21.73)	--	300	100 (33.3)	8 (2.6)	110	26 (23.63)	4 (3.6)
Egg	150	56 (37.3)	12 (21.4)	252	78 (31)	16 (6.34)	96	40 (41.6)	6 (6.25)
Fish									
*T. ilisha*	16	6 (37.5)	--	30	10 (33.3)	1 (3.3)	0	0	--
Prawn	18	8 (44.4)	3 (16.6)	26	16 (61.5)	6 (23)	12	6 (50)	7 (58.3)
*L. rohita*	98	6 (6.1)	2	144	12 (8.3)	2 (1.4)	62	0	--
Fruits									
Apple	24	0	--	32	4 (12.5)	--	80	5 (6.25)	--
Banana	118	48 (40.67)	4 (3.38)	138	56 (40.57)	5 (3.62)	74	30 (40.54)	--
Cucumber	30	6 (20)	--	86	6 (6.97)	--	66	16 (24.24)	--
Guava	22	6 (27.27)	--	36	10 (27.7)	--	65	15 (23.07)	--
Papaya	76	22 (28.95)	2 (2.63)	120	40 (33.3)	2 (1.66)	40	0	3 (7.5)
Pumpkin	64	10 (15.63)	1 (1.56)	140	30 (21.42)	2 (1.42)	50	9 (18)	1 (2)
Grapes	16	4 (25)	--	68	15 (22)	2 (2.94)	62	10 (1.6)	--
Meat									
Beef	18	4 (22.2)	--	68	14 (20.6)	--	0	0	--
Chicken	78	28 (35.9)	--	98	10 (10.20)	3 (3.06)	40	2 (5.0)	--
Mutton	24	0	--	40	6 (15.0)	1 (2.5)	80	10 (12.5)	--
Milk	144	36 (25)	6 (4.16)	304	56 (18.42)	11 (3.6)	106	22 (20.75)	3 (1.88)
Pulses									
*L. culinaris*	96	18 (18.75)	1 (1.04)	142	16 (11.26)	8 (5.63)	64	14 (21.8)	4 (6.25)
*P. mungo*	22	4 (18.1)	1 (4.54)	68	8 (11.76)	5 (7.35)	36	12 (33.33)	2 (5.5)
Vegetables									
Brinjal	62	28 (45.16)	3 (4.83)	152	66 (43.42)	16 (10.52)	40	22 (55)	3 (7.5)
Cabbage	44	6 (13.6)	--	104	12 (11.53)	1 (0.96)	66	18 (27.27)	--
Cauliflower	64	16 (25)	3 (4.6)	152	40 (26.3)	--	68	26 (38.23)	2 (2.94)
Ladyfinger	104	30 (28.8)	2 (1.92)	202	72 (35.6)	4 (1.98)	88	36 (17.25)	1 (1.13)
Spinach	46	18 (39.1)	1 (2.17)	84	20 (23.8)	3 (3.57)	50	10 (20)	1 (2)
Tomato	140	34 (24.3)	2 (1.43)	258	60 (23.25)	8 (3.1)	78	14 (17.95)	--

F indicates total number of females tested; M, total number of males tested.

In the age range 16 to 40 years (male-female ratio, 1:1.19), 33.3% were sensitive to prawn; brinjal, 43.42%; banana, 40.57%; ladyfinger, 35.6%; papaya, 33.3%; wheat, 33.3%; egg, 31%; cauliflower, 26.3%; spinach, 23.8%; beef, 20.6%; milk, 18.42%; lentil (*P. mungo*), 11.76%; *L. culinaris*, 11.26%; and cabbage, 11.53%. The age group 41 to 60 years (male-female ratio, 1:1.04) had skin reactivity to brinjal (55%), egg (41.6%), banana (40.54%), fish (38%), *P. mungo *(33.33%), cabbage (27.27%), wheat (23.63%), *L. culinaris *(21.8%), milk (20.75%), spinach (20%), and tomato (17.95%). Patients younger than 16 years were sensitized to brinjal (45.16%), prawn (44.4%), banana (40.67), spinach (39.1%), egg (37.3%), papaya (28.95%), ladyfinger (28.8%), cauliflower (25%), milk (25%), wheat (21.73%), *L. culinaris *(18.75%), *P. mungo *(18.1%), and cabbage (13.6%).

**Figure 1:**
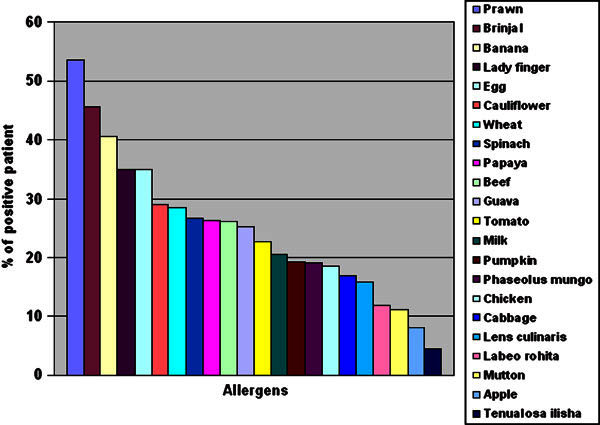
**Frequency of positive response to common food allergens among the respiratory allergy population in Calcutta, India**.

A list of all the allergens and the total number of patients positive to all these allergens are represented in Figure 1 and Table 3.

**Table 3:** Allergens and the Total Number of Patients Positive to These Allergens

Allergens	Total No. Patients Tested	No. Patients Positive (%)
Wheat (cereal)	548	156 (28.47)
Egg	498	174 (34.94)
Fish		
*Hilsa*	46	16 (4.35)
Prawn	56	30 (53.57)
*Rohu*	304	18 (11.84)
Fruits		
Apple	136	11 (8.09)
Banana	330	134 (40.61)
Cucumber	182	28 (15.38)
Guava	123	31 (25.20)
Papaya	236	62 (26.27)
Pumpkin	254	49 (19.29)
Meat		
Beef	69	18 (26.08)
Chicken	216	40 (18.52)
Mutton	144	16 (11.11)
Milk	554	114 (20.58)
Pulses		
Musoor	302	48 (15.89)
Mung	126	24 (19.05)
Vegetables		
Brinjal	254	116 (45.67)
Cabbage	214	36 (16.82)
Cauliflower	284	82 (28.87)
Ladyfinger	394	138 (35.02)
Spinach	180	48 (26.67)
Tomato	476	108 (22.69)

## Conclusions

The present study was undertaken to identify food sensitization in patients aged 5 to 60 years with respiratory allergic symptoms, who reported to our allergy clinic in Calcutta. A total of 684 patients presented a history of allergy to different foods. Among them, 516 (75.44%) patients had a history of allergy to common food allergens such as egg, milk, cereals, and legumes that induce immunoglobulin E-mediated reactions. Among the common food allergens, allergy to egg was highest in the age group 41 to 60 years (41.6%); the frequency of milk allergy was highest in the age group 5 to 15 years (25%), children being the largest consumers of milk; the percentage of hypersensitivity to cereals was highest among patients in their late teens and adults, that is, in the age group 16 to 40 years (33.3%).

These findings are in accordance with other reports on food hypersensitivity in children [4,16,17]. Allergy to legumes (*L. culinaris *and *P. mungo*) was maximum in the age group 41 to 60 years (26%). Legume hypersensitivity on respiratory allergy patients has been similarly reported in India [9,12,18]. It was observed that 315 patients (46.05%) showed allergic reaction to fruits, 64 (9.36%) of them showed a hypersensitive reaction to different kinds of fishes, and 528 patients (77.19%) had positive SPT results for different vegetables considered in our study, which included brinjal, cabbage, cauliflower, ladyfinger, spinach, and tomato.

In our present study, among the allergic population, maximum numbers of patients were hypersensitive to prawn (53.57%) and brinjal (45.67%) (Table 3). Brinjal was found to be most allergenic to patients belonging to the age groups 5 to 15 years (45.16%) and 41 to 60 years (55%), followed by prawn (5-15 years, 44.4%; 41-60 years, 50%). However, patients belonging to the age group 16 to 40 years showed maximum hypersensitivity to prawn (61.5%), followed by brinjal (43.42%). The allergenic reaction to prawn in this age group may be caused by greater consumption of prawn among individuals of this group. Similar results have been reported in patients in Russia, Estonia, and Lithuania [4]. The percentage of allergy to beef was found to be quite high, although it is restricted only among the Muslim and Christian communities of Calcutta because beef consumption is not prevalent among the rest of the population. Thus, we can conclude that apart from age, food hypersensitivity also reflects different genetic factors and variations in cultural and dietary habits of each individual [19].
